# Attentional Focus and Performance Anxiety: Effects on Simulated Race-Driving Performance and Heart Rate Variability

**DOI:** 10.3389/fpsyg.2012.00426

**Published:** 2012-10-19

**Authors:** Richard Mullen, Andrea Faull, Eleri S. Jones, Kieran Kingston

**Affiliations:** ^1^Division of Sport, Health and Exercise, University of GlamorganWales, UK; ^2^Institute of Sport and Exercise Science, The University of WorcesterWorcester, UK; ^3^School of Sport, Cardiff Metropolitan UniversityCardiff, UK

**Keywords:** attention, anxiety, heart rate variability, learning, driving

## Abstract

Previous studies have demonstrated that an external focus can enhance motor learning compared to an internal focus. The benefits of adopting an external focus are attributed to the use of less effortful automatic control processes, while an internal focus relies upon more effort-intensive consciously controlled processes. The aim of this study was to compare the effectiveness of a distal external focus with an internal focus in the acquisition of a simulated driving task and subsequent performance in a competitive condition designed to increase state anxiety. To provide further evidence for the automatic nature of externally controlled movements, the study included heart rate variability (HRV) as an index of mental effort. Sixteen participants completed eight blocks of four laps in either a distal external or internal focus condition, followed by two blocks of four laps in the competitive condition. During acquisition, the performance of both groups improved; however, the distal external focus group outperformed the internal focus group. The poorer performance of the internal focus group was accompanied by a larger reduction in HRV, indicating a greater investment of mental effort. In the competition condition, state anxiety increased, and for both groups, performance improved as a function of the increased anxiety. Increased heart rate and self-reported mental effort accompanied the performance improvement. The distal external focus group also outperformed the internal focus group across both neutral and competitive conditions and this more effective performance was again associated with lower levels of HRV. Overall, the results offer support for the suggestion that an external focus promotes a more automatic mode of functioning. In the competitive condition, both foci enhanced performance and while the improved performance may have been achieved at the expense of greater compensatory mental effort, this was not reflected in HRV scores.

## Introduction

Over the last decade, researchers have demonstrated the beneficial effects of an external focus of attention for the acquisition of motor skills (see Wulf, 2007 for a review). Wulf and colleagues have conducted a series of experiments comparing the relative effectiveness of an external versus an internal focus of attention. An external focus involves directing one’s attention to the effects that body movements have on the environment, while an internal focus is directed at the body movements themselves (Wulf et al., 1998). Using a series of different tasks, Wulf and associates have consistently demonstrated that learning using instructions that encourage an external focus is more effective than learning using instructions that are internally focused.

Within this growing field, an external focus has also been conceptualized in several forms by different researchers. Specifically, researchers have distinguished between distal and proximal external foci. For example, McNevin et al. (2003) used a stabilometer balance task and demonstrated that a distal external focus of attention on balancing platform markers placed 26 cm away from participants’ feet resulted in enhanced learning compared to a proximal external focus that required participants to focus on markers placed immediately in front of their feet, which, in turn, was more effective than an internal focus of attention. It appears that an external focus has more beneficial effects on learning when the direction of focus is remote from the action effects that produce the movement. Despite the support for a distal external focus produced by McNevin et al. (2003), other studies have found contrasting results (e.g., Wulf et al., 2000; Marchant et al., 2007). Subsequently, Bell and Hardy (2009) suggested that one reason for the mixed results reported in the literature might have been due to the different ways in which an external focus has been adopted by researchers. Bell and Hardy suggested that an external focus that directed an individual’s attention toward the outcome or target of a movement, for example the flag in a golf chip shot, or the bullseye in darts, fell outside the limits of Wulf et al.’s original conceptualization of an external focus, which was defined as those instances where a performer’s attention is directed to the effect of the body’s movement on the external environment. As such, distal external foci that direct an individual’s attention toward task performance processes, for example the flight of the ball in a golf chip shot, appear to be more in line with Wulf et al.’s original specification. Using this distinction, Bell and Hardy examined the performance of skilled golfers using a distal external focus on the desired trajectory of the ball after it had left the club face, a proximal external focus on the position of the club face throughout the swing, and finally, an internal focus on maintaining the hinge in the wrists that golfers typically adopt during the swing.

Bell and Hardy also proposed that an external focus of attention might have beneficial effects in high pressure, competitive situations in which performers experience high levels of state anxiety. Two lines of evidence led Bell and Hardy to this conclusion. Firstly, Wulf et al. (2001) demonstrated that adopting an external focus of attention was associated with reduced cognitive demands. Such a reduction enables performers adopting an external focus to allocate attentional resources to deal with the potentially distracting effects of cognitive anxiety and maintain primary task performance. Secondly, Totsika and Wulf (2003) used a transfer test to demonstrate that relative to an internal focus of attention, an external focus results in more robust performance in attention-demanding secondary task conditions. In Totsika and Wulf’s study, participants learned to ride a pedalo using either internal or external focus instructions. The subsequent transfer task involved riding the pedalo while counting backward in threes. At transfer, the external focus group outperformed the internal focus group, supporting the notion that an external focus is associated with reduced cognitive demands.

Building on this earlier work, Bell and Hardy had two aims, to examine (i) the multidimensional nature of an external focus and, (ii) the robustness of the different foci in anxiety-invoking conditions. Bell and Hardy assigned 33 skilled male golfers to one of the three attentional focus conditions, an internal focus on the movement of the arms during the swing, a proximal external focus on the clubface, and distal external focus on the flight of the ball after it had left the clubface. Participants completed five blocks of 10 pitch shots, three in neutral conditions and two in a competitive condition designed to increase state anxiety. Bell and Hardy found that a distal external focus was more effective than either an internal focus or a proximal external focus, regardless of anxiety condition. However, Bell and Hardy compared the relative effectiveness of the attentional focus conditions in the neutral and anxiety-invoking conditions separately. A more complete investigation of the effect of anxiety on the different attentional foci would involve within-subject analyses of participants using internal and external foci across anxiety conditions. In addition, it is not clear that the benefits of a distal external focus would generalize to other tasks and levels of expertise.

More generally, Wulf et al. (2001) proposed the constrained action hypothesis to explain the beneficial effects of external foci. Wulf et al. suggested that attempting to control movements consciously using an internal focus disrupts task execution by interfering with the automatic control processes that normally regulate the movement. In contrast, adopting an external focus promotes more unconscious, fast, and reflexive task processing that is more automatic in nature. While automatic processing is typically seen in expert performers, Wulf and associates have produced three lines of evidence suggesting that relative novices who use an external focus of attention can also produce movements that appear to be more automatic in nature. Firstly, Wulf et al. (2001) asked participants to balance on a stabilometer using either an internal or external focus of attention. Frequency domain analysis of the movement of the stabilometer demonstrated that an external focus encouraged movements that were characterized by faster and more frequent adjustments. The higher frequency adjustments allow the motor system to respond quickly to environmental or within-person perturbations, exploiting the available perceptual-motor degrees of freedom to produce smoother movements (Newell and Slifkin, 1996). In addition, Wulf et al. used probe reaction time to test the prediction that the automatic nature of movements resulting from an external focus would be associated with less consumption of attentional resources, producing faster probe reaction times. As predicted, an external focus resulted in quicker reaction times. The third line of evidence supporting the constrained action hypothesis has been accrued using neuromuscular responses (EMG; e.g., Vance et al., 2004; Zachry et al., 2005). There is now strong support for the suggestion that an external focus generally produces less EMG activity in comparison to internal focus conditions (see Marchant, 2011 for a review)

Studies using psychophysiological measures have also added to the evidence supporting the constrained action hypothesis. For example, Schucker et al. (2009) examined the effect of attentional focus on running economy in experienced athletes using three conditions, an external focus on participants’ surroundings and two internal focus conditions, on running movement and breathing. Oxygen consumption and subjective ratings indicated that the external condition produced movements that were more efficient when compared to both of the internal focus conditions. Lohse and Sherwood (2011) also used a subjective measure of exertion to examine the effect of attentional focus on a muscular endurance task, an isometric wall-sit. An external focus reduced both time to fatigue and perceived exertion. Phasic heart rate (HR) has also been used to examine the costs associated with different attentional foci (e.g., Radlo et al., 2002). To date, however, researchers have overlooked the contribution that tonic measures of HR could make to the assessment of the attentional demands associated with different attentional foci. Heart rate variability (HRV), estimated by spectral analysis of the cardiac signal, is one variable that has been used to index the cardiac activation state associated with attentional demands (Berntson et al., 1997; Fairclough and Mulder, 2011). Spectral decomposition of the HR signal produces periodic components of HRV aggregated within three main frequency bands, which are associated with different functional influences in the modulation of HR. The first of these, the very low-frequency (LF) band (0.02–0.06 Hz), reflects thermoregulatory control (Grossman, 1992); the LF band (0.07–0.14 Hz) is hypothesized to represent the cognitive loading associated with controlled processing (Fairclough and Mulder, 2011); finally, the high-frequency (HF) band (0.15–0.40 Hz) is related to momentary respiratory influences or respiratory sinus-arrhythmia (Grossman, 1992). Of these three bands, the LF band has more consistently responded to a range of manipulations that cause major changes in task structure and induce changes in the mode of operation, as in the shift from automatic to controlled processing (Jorna, 1992; Veltman, 2002). Evidence supporting this suggestion has been demonstrated in several studies that examined mental workload demands during computer-based tasks (Neumann, 2002) or simulations of complex human-machine interactions (Jorna, 1992; Veltman and Gaillard, 1998). In the context of motor performance, Neumann and Thomas (2009) found additional support for sensitivity of the LF band by comparing the cardiac power spectrums of novice and expert golfers. Neumann and Thomas hypothesized that novice performance would be directed using more resource-intensive controlled processing, while that of experts would be under the direction of less demanding automatic processes. Consistent with this prediction, the HRVLF band response indicated that the experts appeared to invest less mental effort in the task. The experts also had lower overall HRs than the novices, also indicative of lower overall effort expenditure. However, Neumann and Thomas’s results should be interpreted with caution as they failed to include resting baseline measures of the cardiac variables. Research in this area is typically conducted using change scores from resting baselines (Mullen et al., 2005), or by including the resting baseline as an additional level in the statistical analysis (Veltman and Gaillard, 1996; Wilson et al., 2007). The absence of any comparative baseline measure makes the interpretation of the HR power spectrum problematic.

While HRV has not been used to examine the cardiac activation states underpinning external and internal foci, it has been used in research examining attention and anxiety effects. For example, Mullen et al. (2005) found no effects of anxiety upon HRVLF in their study that examined whether conscious processing or attentional explanations could best account for performance anxiety effects upon golf-putting performance. While there were no effects of anxiety upon HRVLF, anxiety-related performance impairment was associated with changes in in the HRVHF band, which the authors suggested might be related to changes in breathing-based relaxation strategies. Also using a golf-putting task, Wilson et al. (2007) used HRV in a study to examine psychophysiological responses related to attention and anxiety. They also found that anxiety had no effect upon HRVLF but did report that self-reported mental effort was sensitive to anxiety effects. Specifically, using the Rating Scale of Mental Effort (RSME: Zijlstra, 1993), participants perceived that they invested more effort in task execution when they were anxious. De Waard (1996)also used the RSME and reported that the scale is sensitive to effort related to both controlled processing and also the compensatory effort used by participants to help overcome the threat to performance of increased anxiety (cf. Wilson et al., 2007). Taken together, the results of the Mullen et al. and Wilson et al. studies are inconclusive on the effect of attention and anxiety on HRV, although direct comparisons are difficult due to the different ways in which the cardiac data were collected, pre-processed, and analyzed. Evidently, more research is required to establish how anxiety and attention interact to affect the cardiac activation states that underpin performance.

In terms of attentional focus, examination of HRV could provide additional support for the suggestion that an external focus encourages more efficient automatic processing, while an internal focus is associated with more effortful controlled processing. Adopting an internal focus should result in greater reductions in HRVLF spectral power from baseline relative to an external focus, reflecting the extra mental effort associated with controlled processing. To date, no previous studies have examined HRV as an index of the attentional processing associated with internal and external foci. In addition, apart from Wulf and associates’ original research using a balancing task, researchers have mainly focused upon examining discrete skills such as golf putting and dart throwing in order to examine the phasic response of HR prior to task execution. Therefore, the primary purpose of this study was to use a continuous task, simulated driving, to examine Wulf’s predictions and the pattern of HRV underpinning task execution. We measured inter beat intervals across a period of skill acquisition to examine the extent to which a distal external focus of attention would be associated with lower levels of mental effort, relative to an internal focus condition. We used a distal external focus condition for two main reasons. Firstly, there is growing evidence to support the advantage of a distal compared to proximal external focus (McNevin et al., 2003; Bell and Hardy, 2009) and secondly, based upon a pilot study, we sought to increase the “distance” between the internal and external foci in order to maximize any attentional focus effects (cf. Wulf et al., 2001). We also set out to test the robustness of the acquired skill in a competitive transfer condition designed to increase state anxiety.

During practice, we predicted that participants who adopted an external focus would outperform those who used an internal focus during practice and this performance advantage would be associated with smaller reductions in HRV spectral power from baseline, reflecting the reduced attentional demands associated with more automatic processing. In the competitive condition, we predicted that cognitive state anxiety would increase and that an external focus would enable participants to maintain performance, levels of HRV, and self-reported effort close to those observed in practice, while an internal focus would be associated with impairment of performance and increased mental effort.

## Materials and Methods

### Participants

Sixteen male undergraduate students between 18 and 26 years of age (mean = 19.68 years, SD = 1.82) from a university in the United Kingdom were recruited for the study. Participants reported no experience of the driving game used in the study, had been in possession of a full UK driving license for at least 1 year (mean = 2.21 years, SD = 0.93), and provided informed consent. Ethical clearance was obtained from the university ethics committee.

### Apparatus

Participants completed a driving simulation task using the Gran Turismo^™^ video game (Sony Computer Entertainment America; Foster City, CA, USA) presented on a 32″ screen. Participants controlled the simulator using an analog force feedback steering wheel and pedals and maneuvered the car around the “High Speed Ring” track option in a Mazda MX5 with automatic gear changes. Participants, who all used the driver’s perspective, drove in time trial mode to avoid any confounding effects of other cars that appeared on track in other race modes. HR data were collected using Ag/ACl pre-gelled electrodes attached to three sites on the participant’s chest: the sternum, the lower right rib cage, and the lower left rib cage (V5/V6). Interbeat intervals were determined using a dedicated *R*-peak trigger that detected the QRS complex in the electrocardiogram.

### Design

Participants were tested on three consecutive days. The first 2 days comprised the practice phase of the study, during which participants completed eight blocks of two trials (1 trial = 2 laps). Four blocks were completed on day one and four on day two. The third day consisted of two blocks of two trials completed in a competition condition designed to increase state anxiety. In order to compare the competition condition with a neutral condition, the final two blocks of the practice phase were used as the neutral comparison condition (cf. Liao and Masters, 2002). In total, each participant completed eight blocks of two trials (32 laps) during the practice phase, and two blocks of two trials (eight laps) in the competition condition. Each trial consisted of 24 corners, so in total, participants completed 384 repetitions of the steering task during practice and a further 96 in the competitive condition.

### Measures

#### Performance

Driving performance was assessed using lap times recorded by the simulator. Participants were not informed that lap times were being recorded. Performance was also measured using the number of driving errors made. An error was made if two or more wheels left the track, if the car hit a wall or barrier, or if the car spun.

#### Cardiac variables

Heart rate was recorded throughout practice and competition conditions. Both data collection and sampling epochs were controlled using the same computer clock. To standardize the epoch for spectral analysis, the middle 3 min of each driving block was used. The length of time taken to complete each block ranged from 4.49 to 6.00 min. Artifact correction was conducted according to procedures used by Mulder (1992) and Mullen et al. (2005). For each participant, total artifact time was always less than 5% of total registration time during any session. The artifact-free data were detrended using a smoothness priors based approach (Tarvainen et al., 2002).

Power spectrum densities (PSD) were estimated using autoregressive (AR) methods (Kubios HRV program, Biosignal Analysis and Medical Imaging Group, University of Kuopio, Finland). Compared to fast Fourier transforms, AR algorithms produce a superior resolution, especially in short samples such as those used in the present study. HRV was estimated in the LF (0.07–0.14 Hz) and HF (0.15–0.40 Hz) spectral bands. The PSD for the frequency band is reported in normalized units (ms^2^). The scores used in the analysis of the HRV data are the differences between mean spectral power for the resting baseline and the values obtained in the experimental conditions. The scores represent reductions from baseline; hence a larger value represents a larger reduction from the baseline. For HR, difference scores are again used but here the scores represent increases from baseline.

#### Self-reported effort

Perceived mental effort was assessed using the RSME (Zijlstra, 1993), which has demonstrated acceptable reliability in laboratory (*r* = 0.88) and real-life work settings (*r* = 0.78). This retrospective one-dimensional visual analog scale requires participants to rate how much mental effort they perceived they invested into a task on a vertical scale ranging from 0 (*not at all effortful*), through 115 (*tremendously effortful*), to 150 (*no anchor*). Participants are required to mark the scale at the point that best reflects the amount of mental effort invested in task performance. The RSME was not used during the learning trials as Meijman et al., 1985, cited in Zijlstra, 1993, p. 102) demonstrated that the effects of higher workload as indexed by the RSME, are not immediately apparent but are delayed. Typically, the RSME is administered at the end of a work period. Consequently, we adopted a cautious approach to the use of the RSME and administered the scale following the neutral and competitive conditions only.

#### Competitive state anxiety

State anxiety was measured using the cognitive anxiety subscale of the Competitive State Anxiety Inventory-2 (CSAI-2; Martens et al., 1990). The CSAI-2 is a sport specific, self-report inventory that has been shown to be a reliable and valid measure of cognitive and somatic anxiety and self-confidence. Alpha reliability coefficients range from 0.79–0.90 (Martens et al., 1990). For this study, participant instructions and some of the items were adapted to make them task-specific. For example, “I am concerned about performing poorly” was altered to read, “I am concerned about driving poorly”. Participants rated their cognitive anxiety on a Likert scale ranging from one (*not at all*) to four (*very much so*).

#### Manipulation check

Participants were asked a single question to determine whether they had maintained their assigned focus, requiring a yes or no response.

### Experimental conditions

Participants were randomly assigned to one of two attentional focus conditions and received written instructions detailing the cues that they were required to use while steering around bends. Participants in both conditions were instructed to keep their vision focused on the track at all times during the task. The cues were constructed with the assistance of two BASES accredited sport psychologists in line with driving instruction literature (Senna and Howell, 1993). Our study did not include a proximal external focus group, which would have entailed a focus on the steering wheel, as pilot testing indicated that participants in this condition reported being unable to maintain their focus and continually allowed their attention to drift to their hand movements. In the pilot study, participants who were asked to focus internally on hand movements reported no such crossover focus on the steering wheel.

#### External focus group

Participants were instructed to focus on the planned trajectory of the car through the next bend as they approached it. Participants were asked to use the cue *outside, inside, outside*, which encapsulated the ideal planned movement of the car from the turning in point (on the outside of the track) to the apex of the corner (the inside of the corner) to the exit, or end of the corner (the outside of the track again).

#### Internal focus group

Group members were instructed to focus on using the outside hand to turn into the corner in the most efficient way. For a left hand bend, this meant that the right hand (outside hand) primarily turned the steering wheel, while the left (inside) hand merely followed the movement. Participants were asked to use the cue *outside hand* to guide their hand movements. Importantly, the focus in this condition was on the hand movement and not the steering wheel, which would constitute a proximal external focus.

### Procedure

Participants were asked to abstain from consuming caffeine up to 3 h before attending the laboratory and to refrain from practicing similar tasks between testing sessions. Participants attended the research laboratory individually and were told that the researcher was interested in the effects of concentration on a simulated driving task.

#### Day one

Participants were fitted with the ECG electrodes and then sat quietly for 3 min to stabilize HR before a 6-min resting baseline was recorded. Participants completed five warm up laps, and then read instructions about their attentional cue, which they used for the duration of the study. Participants then completed two warm up laps of the track using their assigned cue before completing the practice trials. Participants were reminded to use their assigned cue before each practice block. On completion of the second acquisition block, participants received a 3-min break. When four acquisition blocks were completed, participants completed the manipulation check.

#### Day two

Preliminary procedures were the same as day one. The middle 3 min of the resting baseline were used for subsequent spectral analysis. HR measured at this stage was used as the resting baseline because participants had completed 1 day of testing and were more likely to be relaxed in the laboratory environment. Participants repeated the procedure from day one but did not complete the familiarization session. During the 3-min break following the second acquisition block, participants completed the cognitive anxiety subscale of the CSAI-2 to establish state anxiety levels in a non-threatening condition. The RSME was administered after the end of the final acquisition block.

#### Day three (anxiety intervention)

Preliminary procedures were the same as day 1 and 2. Participants then received instructions informing them that they were involved in a competition and that they had been assigned to a team. Participants were told that the winning team would be the team who produced the fastest aggregate lap time and that each member of the winning team would win £10. Individual target times were assigned to participants, giving them a “false” time that they were told they had to achieve in order for their team to have a chance of winning the task. The target times were calculated by taking the participant’s fastest lap time from practice minus 1.5 s. Pilot testing had indicated that participants perceived this target as challenging but realistic. In sum, participants perceived the target time as being of both personal and team importance, creating an ego-threatening situation that was likely to increase cognitive state anxiety levels. Following two warm up laps participants completed the cognitive anxiety measure, followed by two blocks of driving. At the end of the last block, participants completed the RSME, and were then thanked for their participation and debriefed about the true objectives of the experiment.

## Results

The data were analyzed using two-factor mixed model analyzes of variance (2 × 8; Focus × Block, with repeated measures on the Block factor and 2 × 2; Focus × Competition, with repeated measures on the Competition factor, for the practice and competition phases, respectively). Significant effects were followed up using Tukey HSD pairwise comparisons.

### Manipulation check

Two participants, one from each group, indicated that they did not use their assigned cue on one or more days of the study. The analysis was run with and without the problematic participants. The results were identical and therefore the full data set is reported here.

### Practice

Mean (±SD) values for performance and cardiac variables for the practice phase are shown in Table 1 and summary statistics can be found in Table 2. For lap times, the significant main effect for Block confirmed that performance significantly improved over practice, indicated by a decrease in lap times. The main effect for Focus approached significance, *p* = 0.06, ${\text{η}}_{\text{p}}^{\text{2}}=$ 0.23, and this was likely to be attributed to the slower times recorded by the internal focus group. The performance improvements were not made at the expense of driving accuracy as the significant main effect for Block for the number of driving errors was also significant. Examination of the means indicated that errors decreased as a function of practice. For both HRVLF and HRVHF, the significant main effect for Focus indicated that the internal focus group recorded larger reductions from baseline compared to the external focus group. For HR, there was also a main effect for Focus, with the internal focus group recording larger increases from baseline compared to the external focus group. The main effect for Block was also significant; however, no consistent patterning of HR response was evident over the practice phase.

**Table 1:** **Means (±SD) for performance and cardiac variables for practice**.

Variable	AFC	Blocks							
		1	2	3	4	5	6	7	8
Lap time	External	88.28 (13.92)	79.32 (14.40)	74.57 (12.53)	75.91 (12.22)	73.39 (9.65)	72.86 (10.05)	70.15 (7.42)	68.81 (6.05)
	Internal	113.76 (22.80)	103.04 (32.04)	99.40 (29.15)	89.33 (23.13)	84.12 (18.63)	87.95 (21.72)	81.92 (18.05)	79.83 (16.70)
Errors	External	5.97 (2.28)	4.44 (2.04)	4.06 (2.34)	3.72 (2.24)	3.22 (1.70)	3.31 (1.70)	2.47 (1.46)	2.19 (1.15)
	Internal	5.44 (3.99)	4.28 (4.16)	3.96 (2.97)	3.28 (3.05)	3.59 (3.71)	2.93 (2.75)	2.34 (2.06)	2.19 (2.21)
HRVLF	External	1841 (1975)	1605 (1333)	1599 (919)	2046 (2581)	1304 (1087)	1270 (1135)	1285 (1254)	1396 (1368)
	Internal	2387 (2040)	3803(2699)	3963 (4638)	3687 (2273)	1733 (1518)	3294 (3052)	1452 (1788)	2774 (3118)
HRVHF	External	1431 (1714)	1319 (1603)	417 (411)	1428 (2030)	1088 (962)	831 (767)	570 (663)	1695 (1865)
	Internal	6227 (9144)	4378 (4509)	3050 (4459)	4484 (5210)	2378 (3219)	2429 (1886)	1004 (948)	2184 (2235)
HR	External	6.76 (12.85)	8.89 (12.07)	0.90 (8.13)	5.89 (10.58)	4.83 (6.31)	4.98 (7.47)	0.58 (6.21)	3.90 (6.92)
	Internal	28.98 (15.73)	34.34 (22.84)	21.39 (11.47)	26.76 (15.51)	35.11 (39.28)	33.76 (38.28)	9.30 (14.10)	34.94 (39.53)

*AFC, Attentional focus condition*.

**Table 2:** **ANOVA summaries for all variables for practice and competition**.

Variable	Practice				Competition		
	df	*F*	${\text{η}}_{\text{p}}^{\text{2}}$		df	*F*	${\text{η}}_{\text{p}}^{\text{2}}$
**LAP TIMES**							
Focus	1, 14	4.12	0.23	Focus	1, 14	4.28*	0.23
Block	2.50, 35.03+	20.68**	0.56	Competition	1, 14	22.14**	0.61
Focus × Block	2.50, 35.03+	2.80	0.17	Focus × Comp	1, 14	0.01	0.001
**DRIVING ERRORS**							
Focus	1, 14	0.02	0.00	Focus	1, 14	0.03	0.00
Block	2.59, 36.19+	13.15**	0.80	Competition	1, 14	1.25	0.08
Focus × Block	2.59, 36.19+	0.21	0.00	Group × Comp	1, 14	0.19	0.01
**HRVLF**							
Focus	1, 14	4.66*	0.25	Focus	1, 14	4.99*	0.26
Block	2.41, 33.81+	1.24	0.08	Competition	1, 14	0.18	0.01
Focus × Block	2.41, 33.81+	0.73	0.05	Focus × Comp	1, 14	0.01	0.00
**HRVHF**							
Focus	1, 14	5.03*	0.26	Focus	1, 14	4.67*	0.25
Block	2.37, 33.21+	2.12	0.13	Competition	1, 14	1.07	0.07
Focus × Block	2.37, 33.21+	1.32	0.09	Focus × Comp	1, 14	1.01	0.07
**HEART RATE**							
Focus	3, 28	5.00*	0.35	Focus	1, 14	3.76*	0.29
Block	1.94, 54.33+	6.52*	0.19	Competition	1, 14	19.21*	0.41
Focus × Block	5.82, 54.33+	2.00	0.18	Focus × Comp	1, 14	1.30	0.12
**SELF-REPORTED EFFORT**							
				Focus	1, 14	0.20	0.12
				Competition	1, 14	18.91**	0.57
				Focus × Comp	1, 14	0.12	0.01
**COGNITIVE ANXIETY**							
				Focus	1, 14	0.04	0.00
				Competition	1, 14	13.02**	0.48
				Focus × Comp	1, 14	0.52	0.04

*significant at p < 0.05; **significant at p < 0.001; +corrected for lack of sphericity using Greenhouse–Geisser epsilon; Comp, Competition; ${\text{η}}_{\text{p}}^{\text{2}}=$ partial eta squared.

### Competition phase

Means for cognitive state anxiety can be found in Table 3. A significant main effect for Competition confirmed that for both groups, cognitive anxiety levels increased following the anxiety manipulation (see Table 2). In terms of performance, the mean lap times for the last two practice blocks (neutral condition) were compared with the mean lap times of the two competition blocks (Table 3). The ANOVA revealed a significant main effect for Competition, indicating that all participants posted faster times in the competitive condition. A significant main effect for Focus was also found, indicating that overall, the external focus group outperformed the internal focus group. There were no significant effects for driving errors. For both the HRVLF and HRVHF data, the analyzes revealed a similar pattern of effects to those found for practice as the internal focus group recorded significantly larger reductions of spectral power from baseline across both the neutral and competition conditions compared to the external focus group (see Table 3). Neither frequency band was affected by the anxiety manipulation. Analysis of the HR data revealed significant main effects for Focus and Competition. For Focus, the internal focus group recorded larger increases from baseline compared to the external focus group. The Competition main effect indicated that HR was higher for both groups in the competitive condition. The significant main effect for Competition for the RSME scores revealed that participants felt that they invested more mental effort into the task during the competition.

**Table 3:** **Mean (±SD) values for all variables for the competition phase**.

Variable		Competition condition	
		Neutral	Competition
Cognitive anxiety	External	15.25 (4.23)	18.25 (5.23)
	Internal	14.13 (3.27)	18.63 (4.03)
Lap time	External	68.81 (6.05)	63.44 (4.28)
	Internal	79.83 (16.71)	74.66 (12.19)
Errors	External	2.19 (1.15)	1.69 (1.63)
	Internal	2.19 (2.22)	1.97 (2.21)
HRVLF	External	1396 (1368)	935 (858)
	Internal	2774 (3118)	2480 (2704)
HRVHF	External	976 (887)	1331 (1064)
	Internal	4245 (5751)	2968 (2199)
HR	External	2.24 (6.24)	18.43 (22.92)
	Internal	22.12 (25.63)	46.69 (30.28)
RSME	External	95.88 (13.64)	112.87 (22.92)
	Internal	104.25 (12.77)	118.75 (16.42)

## Discussion

This study had two main objectives. First, using HRV, we wanted to determine the pattern of mental effort underpinning the acquisition of a simulated race-driving task under external and internal attentional focus conditions. Secondly, we sought to examine the effect of increased cognitive state anxiety on the performance of the driving task and associated levels of HRV. For the practice phase, our results were generally consistent with our hypotheses and those of Wulf and associates (Wulf, 2007), as all participants improved their performance over the acquisition period and those in the external condition outperformed those in the internal condition, although this effect only approached the 0.05 significance level. In addition, HRVLF and HRVHF spectral power were significantly closer to baseline in the external focus condition, indicating that participants used less mental effort than participants in the internal focus condition, supporting the suggestion that externally focused processing may be more automatic in nature. In the competition condition, despite significant increases in cognitive state anxiety, our hypotheses were not supported as both groups significantly improved their performance. In addition, the marginally non-significant performance advantage enjoyed by the external focus group during acquisition was augmented across both the neutral and competition conditions. Similarly, the between group differences in HRVLF, HRVHF, and HR also remained across the neutral and competition conditions. The increases in self-reported mental effort in the competition condition suggest that participants may have made more resources available to assist performance in threatening conditions.

For practice performance, although the main effect for Focus for the lap times only approached the 0.05 significance level (*p* = 0.06), the associated effect size of 0.23 would appear to be of practical significance. As such, the results for the learning phase add support to the robust finding that an external focus of attention enhances task execution, compared to an internal focus (Wulf et al., 2001; McNevin et al., 2003; Wulf, 2007). The results also strengthen the notion that a distal external focus encourages superior performance compared to an internal focus during learning (McNevin et al., 2003) and in high-pressure situations (Bell and Hardy, 2009). In the Bell and Hardy study, the distal external focus was operationalized as a focus on the trajectory of the ball as it left the golf club, a condition similar to the focus upon the anticipated trajectory of the car used in the present study. Despite these similarities, the findings from the Bell and Hardy study were found with skilled participants, whereas the present study used novices. There is some debate in the attentional focus literature regarding the utility of asking novices to use a distal external focus (Wulf, 2007). Wulf suggests that novices may benefit from a more proximal external focus that the learner can more directly relate to the movements that produce an action (cf. Wulf and Su, 2007). We were unable to fully examine this issue, as our study did not include a proximal external focus condition. Clearly, there is further scope to examine the impact of playing experience and ability as moderators of attentional focus effects. Overall, the results presented here add to the generalizability of the distal external focus effects.

Turning to the HRV response, during the learning phase both groups showed reduced power from baseline in the LF band, which supports the notion of increased mental effort associated with task engagement (Jorna, 1992; Veltman and Gaillard, 1998). Furthermore, as predicted, the internal focus group recorded larger LF decreases from baseline compared to the external focus group, indicating that more mental effort was expended. The larger LF response displayed by the internal focus group supports previous evidence that major differences in the mode of operation, such as that found in the distinction between automatic to controlled processing, are associated with reductions in LF spectral power (Jorna, 1992; Veltman, 2002). The internal focus group appear to have engaged more effortful controlled processing in order to support their performance, while the external focus group relied upon processing that could be described as being more automatic in nature. This pattern of findings adds to the evidence base underpinning Wulf et al.’s (2001) constrained action hypothesis.

The differences between the internal and external focus groups in HF variability during practice were not predicted but do correspond to the work of Fairclough et al. (2005), who also found HF reductions from baseline in a high compared to low demand laboratory task. Reduced HF variability is typically associated with suppression of vagal tone (Grossman, 1992). Both attentional focus groups responded with a reduction in HRVHF from baseline; however, this effect was augmented in the internal focus group, in line with the pattern of variability found for the LF spectral band. Reductions in HF variability are associated with reductions in parasympathetic influence, which might account for the increases in HR observed in the internal focus group. This interpretation should be treated with caution, however, as the potential influence of respiration on HF variability must be considered (Berntson et al., 1997). Thus, future research in this area should include measures of respiratory frequency and depth to support inferences regarding HF variability (e.g., Neumann and Thomas, 2009). The HRV responses observed in the present study are the first to examine the pattern of cardiac activity underpinning the use of different attentional foci. Taken together, the findings provide further support for the distinction between controlled and automatic processing implicated by the constrained action hypothesis.

In the competition condition, contrary to our predictions, both groups improved their performance; however, across both neutral and competitive conditions the external focus group maintained their performance advantage over the internal focus group (cf. Bell and Hardy, 2009). The performance improvements we found contrast with the deficits reported by Totsika and Wulf (2003) in their pedalo task; however, a direct comparison is difficult as Totsika and Wulf used speed and attentional load to increase pressure, they did not set out to examine the effects of competitive state anxiety upon performance.

The performance improvements recorded in the competition condition are a common feature of anxiety research, supporting the often adaptive nature of the cognitive anxiety response (cf. Eysenck et al., 2007; Cheng et al., 2011). Eysenck et al.’s Attentional Control Theory (ACT) offers the best explanation of improved performance in high anxiety conditions. Eysenck et al. suggest that the potentially negative effect of increased cognitive anxiety can be offset by increases in effort. The additional compensatory effort helps maintain or improve performance effectiveness, but at the expense of processing efficiency. This appears to have been the case in the present study, as faster driving times in the competition condition were accompanied by increases in self-reported effort (cf. Williams et al., 2002; Wilson et al., 2007), indicating that the improvements in performance were achieved at the expense of processing efficiency. Although De Waard (1996) had previously found that the RSME was sensitive to both types of mental effort; our findings suggest the self-reported effort may reflect changes in compensatory, but not task-related effort. Despite the criticisms leveled at self-report measures in some quarters (Nisbett and Wilson, 1977), such measures appear to remain important. As Vicente et al. (1987) note, ‘If a person feels loaded and effortful, he is loaded and effortful, whatever the behavioral and performance measures may show’ (p. 175).

The increases in performance and the RSME scores were not accompanied by increases in HRV. Previous studies implicating the HRVLF band as an index of effort expended in response to increased higher state anxiety have also failed to find any significant differences between low and high anxiety conditions (Mullen et al., 2005; Wilson et al., 2007), suggesting that the HRVLF band is limited to indexing increases in mental effort that accompany major differences in task processing, such as those found between external and internal attentional foci. Such a distinction has also been made in the HRV literature. For example, Fairclough and Mulder (2011) noted that experimental cardiovascular effects related to compensatory mechanisms, such as those used to cope with increased state anxiety, are far less clear than those related to task-related effort. As Mulder (1992, p. 211) suggested, ‘Despite the fact that optimal task performance surely requires more (compensatory) effort, it cannot be stated that such an effect appears as a greater reduction of HRV’. It appears that neither the LF nor the HF responses are suitable indices of the compensatory effort that accompanies increased cognitive anxiety. In such situations it is possible that any anxiety-related changes in HRVLF may be masked by the impact of sympathetic responses to increased cognitive anxiety as spectral power in the LF band reflects both sympathetic and parasympathetic activity (Berntson et al., 1997).

One further factor to consider is the strength of the anxiety manipulation. Although the cognitive anxiety scores increased significantly in the competition condition and are comparable with similar studies, the means were well below those reported by athletes in competitive situations (cf. Williams et al., 2002; Mullen et al., 2005). It is possible that larger increases in cognitive anxiety might result in different performance and cardiac effects.

A number of limitations were also evident in the present study. Firstly, as one reviewer of this study noted, our attentional focus instructions lack symmetry. Ideally, external and internal focus instructions should only differ by one or two key words that bias attention externally or internally (cf. Wulf, 2007). Our selection of attentional focus instructions was based upon race-driving instructional literature in an attempt to ensure ecological validity. Future research should seek to use instructions that are less complex and similar in construction to secure greater internal validity. In addition, we did not attempt to control visual attention during the task. As a result, it is possible that participants could have directed their visual attention to their hands, potentially explaining their inferior performance. We do not believe that this was the case in the present study as shifting visual attention to the hands would potentially lead to serious steering faults resulting in increased errors. As the number of errors recorded by the external and internal focus groups did not differ throughout any phases of the study, we do not believe that the internal focus instructions caused a shift in visual attention. We cannot rule out this possibility, however, and the issue could be resolved by using eye movement recording in future research. We did not include a control condition. Operationalizing a control or no instruction condition is problematic as participants will develop and implement their own strategies, which may be internally or externally focused, or a combination of both types of focus. Our decision not to include a control condition was also based on previous research, which has indicated that participants in control conditions consistently produce identical results to those in internal focus conditions (see Wulf, 2007, p.58). Although small, the group size of eight is identical to that used in similar studies in which participants are asked to perform relatively lengthy periods of skill acquisition (e.g., Masters, 1992; Hardy et al., 1996). Despite this similarity, the small sample size will have had a negative impact upon statistical power. Our inclusion of a manipulation check was an improvement on previous research comparing external and internal attentional foci that has failed to perform such checks. However, our check was not as extensive as those adopted by others (Marchant et al., 2007; Bell and Hardy, 2009) but despite this shortcoming, we can be more confident that the observed effects were the result of successful attempts to control the direction of attentional focus. The generalizability of the findings is also limited by the sample of male undergraduate, sports science students who were novices at the driving task. However, the pattern of results obtained corresponds closely to those reported by Bell and Hardy (2009), suggesting that the advantages of a distal focus of attention might be consistent across tasks.

In terms of practical implications, several aspects of the data are notable. Most important is the need for instructors to be aware of the potentially negative effect that verbal instructions relating to internal processes can have on performance. Moreover, the findings from this study suggest that performers should be encouraged to focus their attention distally. Such advice is important when first learning a skill as the positive effects of a distal external focus are apparent early in learning, and also when that skill is subsequently deployed in competitive situations, where cognitive anxiety can have potentially debilitating effects.

To conclude, the data reported here clearly support the utility of a distal external focus as a means of helping learners acquire race-driving skills and deploy those skills effectively in competitive conditions in which cognitive state anxiety is elevated. Our findings with novice participants add weight to those of Bell and Hardy, who established that a distal attentional focus was also effective under conditions of high anxiety for more skilled participants. The results extend previous research because this was the first study to include HRV as an index of the mental effort associated with the different attentional foci. The pattern of HRV indicated that the performance advantage conferred by an external focus is accompanied by a concomitant decrease in mental effort relative to an internal focus.

## Conflict of Interest Statement

The authors declare that the research was conducted in the absence of any commercial or financial relationships that could be construed as a potential conflict of interest.
